# Up regulated virulence genes in M. tuberculosis H37Rv gleaned from genome wide expression profiles

**DOI:** 10.6026/97320630017608

**Published:** 2021-06-30

**Authors:** Rohini Kumari, Pramod Katara

**Affiliations:** 1 Computational Omics Lab, Centre of Bioinformatics, University of Allahabad, Prayagraj, Uttar Pradesh - 211002 India

**Keywords:** Tuberculosis, expression profiling, virulence genes

## Abstract

Identification of up regulated virulence genes in M. tuberculosis H37Rv using genome wide expression profiles is of interest in drug discovery for the disease. Hence, we report 17 up-regulated PPIN (Protein-Protein Interaction Network) enriched potential virulence linked genes using expression data available at the Gene Expression Omnibus (GEO) database for further consideration.

## Background:

M. tuberculosis H37Rv causes tuberculosis is a pathogen of global interest [1]. Drugs (isoniazid, rifampicin, amikacin, capreomycin, kanamycin, etc.,) are effective in controlling the disease. However, the emergence of drug resistance tuberculosis (DR-TB) is a known paradox in this context [2]. India, China, and the Russia share largest numbers of MDR-TB cases (47% of the global total) with increased mortality [1,3]. The M. tuberculosis genomes are available for multiple strains. The genome for the most explored strain H37Rv is known since 1998 [4]. It is of interest to explore the genome data to glean valuable information on the disease causing virulence factors. Expression profiling using data at the Gene Expression Omnibus (GEO) helps to get clues for gene function [5,6]. Therefore, it is of interest to document data on the identification of virulence genes in M. tuberculosis H37Rv using genome wide expression profiles.

## Materials & Methods:

### Selection of gene expression data:

cDNA gene expression data for M. tuberculosis H37Rv was downloaded from the Gene Expression Omnibus (GEO, https://www.ncbi.nlm.nih.gov/geo/) database. Considering the objective of the work three-experiment series GSE93316 (20 GSM; source - pulmonary tuberculosis patients [7]), GSE49760 (24 GSM; source - whole blood from HIV- and HIV+ [8]), and GSE58466 (6 GSM; source - Replicating in Human Type II Alveolar Epithelial A549 cell line [9]) were used, all these three data series are based on the same platform, i.e., GPL4057.

### Data normalization and filtration:

High throughput data need to be pre processed and normalized. Data was directly normalized using the data transformation (log2) GEO2R application at NCBI. Data filtration was done for the presence of empty/duplicated and incomplete sets manually [10].

### Expression profiling:

The fold change (FC) parameter was used to select differentially expressed genes, further genes with less than two FC was filtered out from the further analysis. Grouping of similarly expressed genes, hierarchical clustering with average linkage was completed using the cluster software. In the end, for visualization of hierarchical clustering, Z-score (+/- 4) based Heatmap was developed through heatmapper (http://www.heatmapper.ca/).

### Virulence gene enrichment using the PPIN analysis:

**Figure 1:**
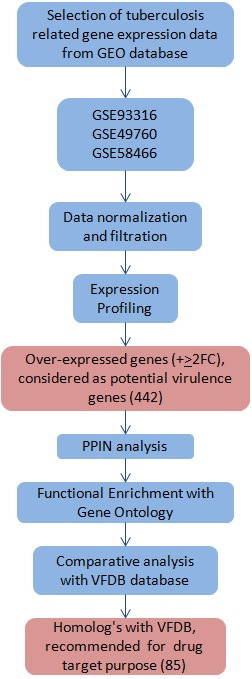
Flow chart, describing complete flow of the work

The systemic view of functional linkage among the predicted genes and their protein-protein interaction network was drawn. For PPIN purpose protein IDs of all genes were used as an input for STRING database (https://string-db.org/), which offer PPIN based functional enrichment analysis. Gene ontology terms for molecular function, cellular component, and biological process were also observed for complete set genes, GO terms with FDR < 0.001 were considered for enrichment. To observe the sequence-based similarity of predicted and reported virulence genes, sequence alignment (blast) with VFDB database done (Figure 1).

## Results and Discussion:

**Figure 2:**
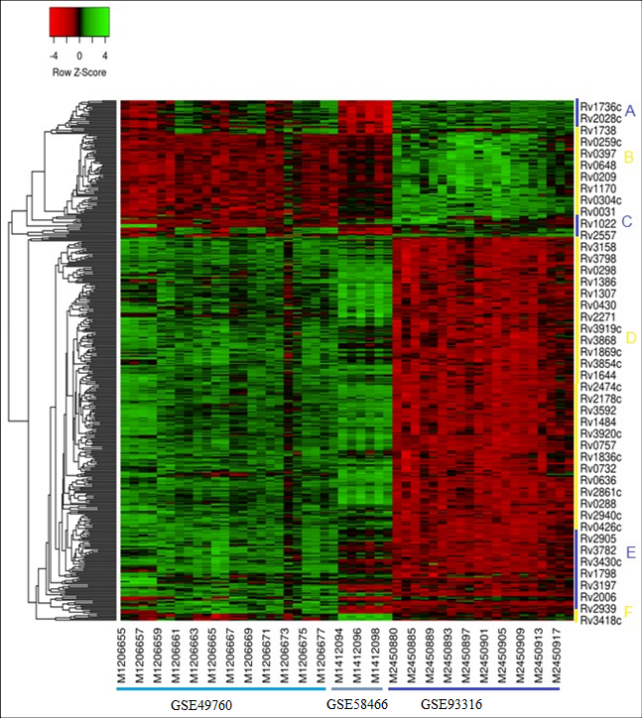
A heat-map for hierarchical clustering of over expressed genes in three different datasets showing gene expression patterns of differential expressed genes of M. tuberculosis. We show the co-expressed genes are clustered in six (A-E) major clusters.

Transcriptome analysis helps to deduce genes affected by a process involved in particular conditions using expression data [11]. Hence, differential gene expression analysis was completed to glean genes with greater than two-fold [12]. Over expression of the genes indicates that they are actively involved in the pathophysiology of tuberculosis during the infection process. Genes with over expression of at least 70% GSM were considered as disease-related genes (442 in numbers). Gene-cluster, hierarchical clustering was done with an average linkage distance. Z-score-based heat-map of hierarchical clustering and across the experiment (GSE) was also completed. This indicates that all the up regulated DEG genes show considerable variability in their gene expression patterns in all datasets grouped in six key clusters (A-E, Figure 2).

Heatmap shows that all genes almost show similar expression variation in GSE49760 and GSE58466, which shows the gene expression in 'whole blood from HIV +/- patients' and H'uman Type II Alveolar Epithelial A549 cell line.' Interestingly, GSE93316, which characterize expression from pulmonary tuberculosis patients, shows an almost opposite expression pattern compare to above mention GSE's. The difference in the sample source, as GSE9336, utilized the sample from patients and GSE49760 and GSE58466 took the transcriptome sample from lab sources, i.e., cultured blood and cell lines, thus differ in the biological milieu [13]. Functional association among the predicted genes was observed through protein-protein interaction network (PPIN) provided by STRING. Graphical representation of the predicted PPIN provides a weight-based functional linkage among the given proteins and helps to understand the systemic view of biological processes [14].

**Figure 3:**
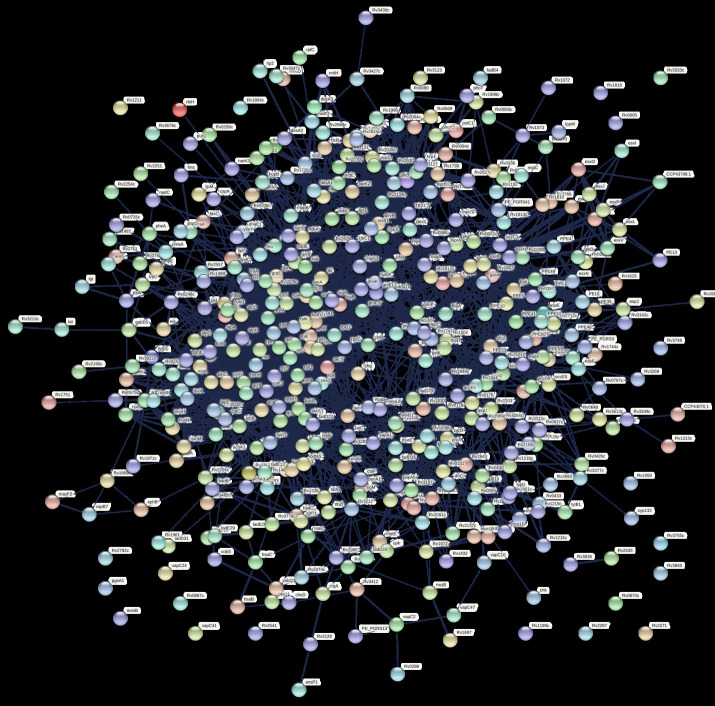
Protein-protein interaction network between potential virulence and associated gene product of M. tuberculosis. Nodes represent proteins and edges represent association.

Network statics of the predicted PPIN shows 2938 edges between 440 protein nodes with an average of 13.3 degrees per node (Figure 3). As the PPIN is a scale-free network whose degree distribution follows power low, where few nodes show very less degree and few are with very high [15]. Predicted network visibly present that network-nodes shows a range of degree distribution which ranging from 01 to 67. The predicted network is also showing the presence of multiple sub-networks, out of them, four are very dense and show high order functional linkage among the member nodes. In total 21 nodes are there in a network with > 50 degrees, top of them are Rv3418c (67), Rv1307 (66), Rv0732 (61), and Rv0704 (60). Gene ontology results from STRING were also observed for the mining of knowledge for considered proteins; all three types of gene ontology have been observed for 442 genes. Molecular function ontology, in total, provides 20 function terms with FDR < 0.001; the most observed gene counts have belonged to binding [protein binding, rRNA binding, enzyme binding, RNA binding, ribonucleotide binding, zymogen binding and cyclic compound binding]. Other important terms belong to transporter activities (proton transmembrane transporter activity; proton-transporting ATP synthase activity, rotational mechanism; active transmembrane transporter activity; transmembrane transporter activity; ATPase-coupled transmembrane transporter activity; inorganic molecular entity transmembrane transporter activity). As Molecular functions generally correspond to activities that can be performed by individual gene products, i.e., a protein/ RNA, here it is clear that these predicted virulence genes mainly belong to binding and transportation [16]. Cellular component terms indicate that predicted genes mainly performed their function in 17 components. The major cellular component term, which shared by more than 100, genes are cell periphery, plasma membrane, external encapsulating structure, cell wall, extracellular region, and cytoplasm. Biological process terms indicate that predicted virulence genes are mainly involved in 21 processes. The most common observed terms are cellular process, growth, potential metabolic process, and response to abiotic stimulus, metabolic process, and transport. All these processes process are very crucial and well reported for their connection with infection and pathogenecity [17-20]. To get more functional insights about the genes, their term description has been mined which more clearly present the functional aspects of the genes (Table 1 - see PDF). Overall 17 terms were observed for the considered genes, as observed through gene ontology, mainly these terms belong to secretion, binding, and transport. All these three terms are well known to relate to microbial pathogenicity, interestingly out of all, 27 genes are already linked to virulence [21,22].

On the basis of differential gene expression followed by Gene ontology and annotation, it is hypothesized that considered genes participate in disease physiology. Gene ontology and annotation terms are not available for all considered genes. In such conditions, sequence homology-based comparative analysis with VFDB database, collections of virulence genes across different pathogens add credence in predicted virulence genes [23]. For this purpose, all genes were compared with this database for their sequence-based similarity search. VFDB core datasets were chosen as target databases for comparison. Result analysis of sequence comparison shows that out of 442 considered genes 27 are already documented in VFDB database and 58 shows considerable similarities with >80% sequence coverage.

## Conclusion:

We report 17 up regulated PPIN (Protein-Protein Interaction Network) enriched virulence linked genes using expression data available at the Gene Expression Omnibus (GEO) database for further consideration in the context of M. tuberculosis infected DR-TB, MDR-TB, and XDR-TB.
